# Public health dentists in maternal and child oral health in India: workforce roles, systemic barriers, and policy implications from a mixed-methods study

**DOI:** 10.3389/froh.2026.1839520

**Published:** 2026-05-12

**Authors:** Omkar Shinde, Vittaldas Shetty, Vikram Garcha, Kadambari Ambildhok, Vineet Vinay, Abhijit Shinde, Firas Elmsmari, Ajinkya M. Pawar

**Affiliations:** 1 Department of Public Health Dentistry, Sinhgad Dental College and Hospital, Pune, India; 2 Shree Gajanan Dental Clinic, Thane, India; 3 Department of Clinical Sciences, College of Dentistry, Ajman University, Ajman, United Arab Emirates; 4 Center of Medical and Bio Allied Health Sciences Research, Ajman University, Ajman, United Arab Emirates; 5 Department of Conservative Dentistry and Endodontics, Nair Hospital Dental College, Mumbai, India

**Keywords:** child health, dental public health, health promotion, health services research, maternal health services, oral health

## Abstract

**Background:**

Maternal and child oral health (MCOH) plays an important role in preventing early childhood caries and improving pregnancy outcomes. Despite global recognition of this link, India has no formal incorporation of oral health into maternal care. Public Health Dentists may be well-positioned to address this gap; however, their roles within maternal and child oral health services have received limited attention. The present exploratory workforce study, therefore examined the perceptions, roles, and systemic barriers experienced by Public Health Dentists in India.

**Methodology:**

A mixed-methods study design was employed. Quantitative data were collected from 150 Public Health Dentists between February and June 2025 using a structured questionnaire that underwent a validation process. This phase was followed by in-depth qualitative interviews with 25 participants to obtain a more detailed understanding of their experiences. Quantitative data were analysed using chi-square tests and multivariable binary logistic regression, while qualitative data were analysed through thematic analysis. The findings were incorporated using triangulation.

**Results:**

A large proportion of respondents (85%) reported being confident in providing oral health education to pregnant women; however, 60% indicated that they had not received formal training in maternal and child oral health. The most commonly reported barriers include limited awareness among patients (40%), financial constraints (25%), and prevailing cultural beliefs (20%). Participants practicing in rural settings reported greater challenges related to accessibility and health literacy. Qualitative findings further highlighted the educator and advocacy roles of Public Health Dentists, while also emphasising systemic limitations and the need for stronger interdisciplinary collaboration. Formal training was significantly associated with higher confidence in service delivery (*p* = 0.002).

**Conclusion:**

The findings suggest that the Public Health Dentists may currently be underutilised in maternal and child oral health services, largely due to structural and training-related limitations. Strengthening the integration of oral health within national maternal health initiatives, including the Reproductive, Maternal, Newborn, Child and Adolescent Health (RMNCH + A) programme, together with competency-based training and improved interprofessional collaboration, could help enhance service delivery and address existing gaps in maternal and child oral healthcare in India.

## 1 Introduction

Oral health is an essential element of general well-being that contributes not only to adult health but also to the health of future generations (1). Maternal periodontal disease has been linked to preterm birth, low birth weight, and early childhood caries, highlighting the importance of maternal oral health for both pregnancy and child outcomes (2). Early childhood development during pregnancy and the early postnatal period plays an important role in shaping long-term oral health outcomes (3). Maternal oral health may also influence the vertical transmission of cariogenic bacteria to infants, which underscores the importance of timely preventive interventions (4). Increasing maternal oral health awareness during prenatal and postnatal periods can prevent early childhood caries and enhance oral health behaviours for both mothers and children (5, 6).

Maternal and Child Oral Health (MCOH) is generally described as a continuum of care that includes oral health education, disease prevention, risk assessment, and appropriate interventions for pregnant women and young children (7). In recent years, MCOH has increasingly been recognised as a priority area within public health frameworks (8). Preventive approaches such as anticipatory guidance, fluoride exposure, and nutritional counselling have demonstrated considerable potential in reducing the risk of early childhood caries (9, 10). Within this context, Public Health Dentists may play a key role in integrating oral health into primary healthcare systems, addressing structural barriers, and improving access to care for mothers and young children (11, 12). In addition to clinical and community roles, they may also contribute to policy advocacy, as illustrated by initiatives reported in countries such as South Africa, Brazil, and Indonesia (13).

Despite ongoing efforts to improve maternal and child oral health (MCOH), comprehensive and integrated strategies remain limited in many parts of the world. Some countries have implemented notable initiatives. For instance, Australia's National Oral Health Plan and Japan's 8020 program demonstrate how intersectoral collaboration can support maternal and child oral health services (14). In contrast, many low-and middle-income countries (LMICs) continue to face persistent challenges, including restricted access to services, shortages in the oral health workforce, and limited public awareness, particularly in rural areas (15, 16). In addition, national health surveillance systems in several settings do not routinely capture information on maternal oral health status or access to related services (17). Previous research has largely focused on maternal knowledge, attitudes, practices, and oral health literacy. However, comparatively little attention has been given to understanding the role and challenges of Public Health Dentists within MCOH service systems (18, 19)

Ongoing disparities in oral health outcomes further highlight the importance of examining systemic factors. Barriers related to workforce capacity, limited resources, and weak intersectoral coordination are often more pronounced in low-resource environments and tend to disproportionately affect vulnerable populations (13). Nevertheless, the specific challenges encountered by Public Health Dentists in delivering maternal and child oral health services remain insufficiently documented.

In India, maternal and child oral health (MCOH) remains a significant yet under-recognised public health concern. Epidemiological evidence indicates a high burden of untreated dental caries among children, with early childhood caries prevalence reported to range between 40% and 70% in different regions. Additionally, studies have demonstrated limited oral health awareness and low utilisation of dental services among pregnant women in India (8, 18). Despite the integration of maternal and child health services under national programs such as RMNCH + A, oral health is not routinely incorporated into antenatal care. This gap is further compounded by disparities in access to care, particularly in rural and underserved populations. These findings highlight the need for greater integration of oral health within maternal healthcare frameworks in India.

Currently, there is no mixed-method study on Public Health Dentists' roles, experiences, and barriers in maternal and child oral health delivery in low- and middle-income settings such as India. Therefore, this exploratory workforce study aimed to examine the roles of Public Health Dentists within maternal and child oral health (MCOH) services in India, with a specific focus on identifying structural and systemic barriers influencing service delivery. In addition, the study sought to develop an integrated conceptual framework based on mixed-method findings to better understand workforce challenges and inform policy and programmatic improvements.

A mixed-methods approach was employed to enable a comprehensive assessment of workforce-related challenges. The quantitative component allowed identification of patterns, associations, and predictors of confidence and service delivery, while the qualitative component provided in-depth contextual insights into underlying reasons, perceptions, and real-world experiences that could not be captured through structured survey data alone. The integration of both components facilitated a more robust understanding of systemic barriers and strengthened the interpretation of findings through methodological triangulation.

## 2 Material and methods

### 2.1 Study design and setting

**Figure 1:**
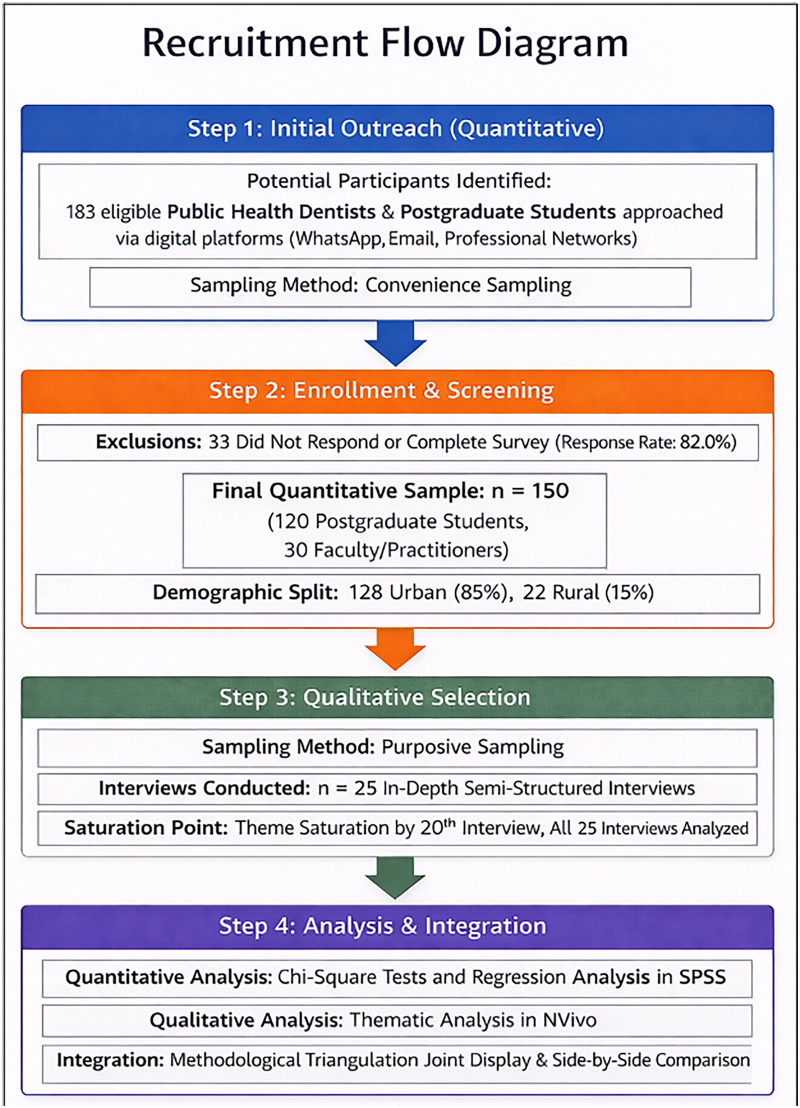
Recruitment flow diagram illustrating participant identification, sampling process, response rate, and integration of quantitative and qualitative phases in the mixed-methods study.

This mixed-method study was carried out at the Department of Public Health Dentistry, Sinhgad Dental College and Hospital, Pune, from February 2025 to June 2025. The study commenced with a quantitative, cross-sectional analysis. The participant recruitment process is illustrated in Figure 1. This study followed the STROBE checklist for cross-sectional studies, the COREQ guidelines for qualitative research, and the GRAMMS framework for mixed-methods studies.

### 2.2 Study population and sampling

Participants were recruited through convenience sampling because a centralized national registry of Public Health Dentists is not available, and the workforce is widely dispersed across India. Considering the exploratory objective of examining workforce perceptions and identifying systemic barriers, recruiting readily accessible participants through professional networks and digital platforms was considered appropriate. Although this approach may limit representativeness, it is frequently used in exploratory workforce studies to generate preliminary evidence and guide future nationally representative research. The questionnaire link was distributed electronically to 183 eligible Public Health Dentists and postgraduate students in Public Health Dentistry across India through professional networks, email groups, and social media platforms, including WhatsApp. Two reminder messages were sent at one-week intervals to improve participation. Participants were recruited from multiple regions across India through professional networks and academic institutions. However, the sample was predominantly drawn from urban and academic settings, which may limit representation from remote and underserved regions.

A total of 150 Public Health Dentists and postgraduate students in Public Health Dentistry were included in the study. Of the 183 participants invited, 150 provided complete responses, corresponding to a response rate of 82.0%. All completed questionnaires met the inclusion criteria and were included in the final analysis. The sample was determined using the formula for estimating a population proportion, assuming a 50% response distribution, 95% confidence level, and 8% margin of error, yielding a minimum of 150 participants. Ethical approval was obtained from the Institutional Ethics Committee of Sinhgad Dental College and Hospital, Pune (IEC Approval number – SDCH/IEC/OUT/2026/34). All participants provided written informed consent before enrolment.

The study included postgraduate students of Public Health Dentistry as well as practising Public Health Dentists who were willing and capable of completing the questionnaire independently. Participants who did not provide consent or were unable to complete the questionnaire were excluded from the study. To ensure anonymity, detailed geographic identifiers at the state level were not collected. Instead, respondents were asked to indicate whether their primary practice setting was located in an urban or rural area. Following consent, participants proceeded to complete the questionnaire and were also informed that they could be contacted for a subsequent in-depth interview.

To ensure data validity and that responses were obtained from eligible participants, several measures were implemented. The survey link was distributed primarily through professional networks, institutional groups, and verified academic channels restricted to Public Health Dentists and postgraduate students. Participation required informed consent, and respondents were asked to confirm their professional status before proceeding. Duplicate responses were minimised by restricting one response per device and reviewing entries for completeness and consistency. Incomplete or inconsistent responses were excluded during data cleaning. These steps helped improve the authenticity and reliability of the collected data despite the use of digital distribution methods.

### 2.3 Study instrument

A structured, self-administered questionnaire (Supplementary File) was designed to evaluate the knowledge, attitudes, involvement, and perceived barriers of Public Health Dentists regarding Maternal and Child Oral Health (MCOH). The questionnaire contained 22 items organised into three sections. Section I collected demographic information, including age, gender, designation, years of professional experience, practice location, and participation in public health programs. Section II included 15 multiple-choice questions intended to assess knowledge of key MCOH concepts such as maternal oral health during pregnancy, anticipatory guidance, appropriate timing of the first dental visit, vertical transmission of cariogenic bacteria, and the integration of oral health within national health programs. Section II focused on respondents' attitudes, confidence levels, practices, and perceived barriers related to MCOH. This section incorporated Likert-scale questions along with dichotomous and open-ended items. The final question provided participants with an opportunity to report any additional challenges while delivering MCOH services.

### 2.4 Questionnaire validation

The questionnaire was subjected to a multistep validation process that included assessment of content validity, face validity, construct validity, and reliability. Five subject experts from the Department of Public Health Dentistry and Pediatric and Preventive Dentistry used a 4-point scale to assess questionnaire items for relevance, clarity and alignment with study objectives. Item Content Validity Index (I-CVI) and Scale Content Validity Index (S-CVI) were estimated, and items with I-CVI < 0.78 were modified or removed. Face validity was examined through pilot testing with 10 postgraduate students of Public Health Dentistry, who received the questionnaire for clarity, readability, and overall flow.

Construct validity was evaluated using Exploratory Factor Analysis (EFA) with Principal Component Analysis and varimax rotation. The Kaiser-Meyer-Olkin measure of sampling adequacy was 0.79, while Bartlett's Test of Sphericity showed statistical significance (*χ*^2^ = 804.12, *p* < 0.001), confirming that the data were appropriate for factor analysis. Three factors with eigenvalues >1 were identified, accounting for 68.2% of the total variance. Cronbach's alpha was used to determine reliability, and values above 0.70 were regarded as acceptable. The overall Cronbach's alpha was 0.81, showing strong internal consistency.

### 2.5 Qualitative component: in-depth interviews

The qualitative component consisted of face-to-face, semi-structured interviews with 25 Public Health Dentists selected through purposive sampling. This sampling approach was used to intentionally include participants with varied experiences, allowing a broader exploration of workforce-level challenges. Participants differed in geographical location (urban or rural), institutional setting (government or private), and years of professional experience.

Participants for the qualitative interviews were selected using purposive sampling to ensure maximum variation in key characteristics, including practice setting (urban/rural), type of institution (government/private), and years of professional experience. Among the 25 participants, 16 (64%) were from urban settings and 9 (36%) from rural areas, while 14 (56%) were postgraduate trainees and 11 (44%) were practising dentists or faculty members. In terms of experience, 12 participants had <5 years, 8 had 6–15 years, and 5 had >15 years of experience. This variation ensured representation of diverse perspectives within the workforce.

All interviews were carried out by a single interviewer trained in qualitative interviewing techniques, with each interview lasting approximately 25–30 min. Using purposive sampling helped ensure that the data reflected a range of perspectives relevant to maternal and child oral health (MCOH). The interviews focused on participants' views regarding their roles, perceived barriers, professional experiences, and suggested improvements. Participants were informed that the interviews were conducted for research purposes to examine challenges and experiences related to maternal and child oral health. Audio recordings of the interviews were processed using Audacity software (Audacity® version 3.7.6; The Audacity Team and Muse Group) and subsequently transcribed verbatim.

### 2.6 Data analysis

#### 2.6.1 Quantitative analysis

Statistical analysis was carried out using the IBM Statistical Package for Social Sciences (SPSS), version 21. Descriptive statistics, including frequencies and percentages, were calculated to summarise the demographic characteristics of the participants. The Chi-square test was used to examine the association between categorical variables, such as confidence in providing maternal oral health education, perceived barriers, training needs, and urban-rural differences. In cross-sectional survey research, the chi-square test is effective for analysing categorical variables.

#### 2.6.2 Qualitative analysis

Thematic analysis was conducted using Braun and Clarke's six-step framework: familiarisation, coding, theme development, reviewing, defining, and reporting (20). This method was chosen because it supports inductive theme development and is the most widely accepted approach in qualitative public health research. Two investigators independently reviewed the transcripts and resolved discrepancies through discussion. NVivo (QSR International, Version 13) was used to assist with systematic coding, data organisation and identification of patterns. NVivo software was used because it enhances analytical transparency and supports rigorous qualitative data management.

### 2.7 Integration and triangulation

Line-by-line coding, inductive categorisation, and disconfirmatory evidence searching were conducted. Theme saturation was reached by the twentieth interview; however, all 25 interviews were included in the analysis to ensure representation across different practice settings. From the analysis, four major themes were identified: the perceived role of Public Health Dentists, barriers to service delivery, training and professional development, and strategies for improvement.

Triangulation was implemented in two ways: first, through data triangulation by including participants from diverse geographic and institutional contexts, and second, through methodological triangulation by integrating quantitative and qualitative findings. Survey data from the quantitative phase and interview data from the qualitative phase were initially analysed separately. The results were then compared side by side to identify areas where statistically significant quantitative findings corresponded with emerging qualitative themes. A joint display table was subsequently created to connect statistically significant urban-rural differences in reported barriers with supporting qualitative excerpts, thereby facilitating integration of findings from both components of the study.

## 3 Results

### 3.1 Quantitative findings

A total of 150 participants were included in the study, of whom 70% were women and 30% were men. Most respondents (80%) were postgraduate students in Public Health Dentistry, whereas 20% comprised faculty members or practising Public Health Dentists. With regard to professional experience, 65% had less than five years of experience, 25% reported six to fifteen years, and the remaining 10% had more than sixteen years. The majority of participants (85%) were based in urban settings, while 15% were practising in rural areas. Overall, the sample largely represented an early-career, urban-based workforce, and the findings therefore predominantly reflect the perspective of postgraduate trainees (Table 1).

**Table 1:** Demographic and practice (geographic) characteristics of participants.

Variable	Category	Frequency (*n* = 150)	Percentage (%)
Gender	Female	105	70%
	Male	45	30%
Education Level	Postgraduate Student	120	80%
	Faculty/Public Health Dentist	30	20%
Years of Experience	<5 years	97	65%
	6–15 years	38	25%
	>15 years	15	10%
Practice Setting	Urban	128	85%
	Rural	22	15%

In terms of professional roles, most participants (90%) reported involvement in planning preventive programs, whereas only a small proportion (10%) indicated performing clinical procedures (*χ*^2^ = 12.4, *p* = 0.002). Most respondents (85%) were confident in educating pregnant women (*χ*^2^ = 8.6, *p* = 0.013), and 75% reported confidence in providing prenatal oral health care (*χ*^2^ = 6.2, *p* = 0.045) (Table 2).

**Table 2:** Role of public health dentists in maternal and child oral health.

Response	Frequency (*n* = 150)	Percentage (%)	Chi-square (*χ*^2^)	*p*-value
Primary Role				
- Designing preventive programs	135	90%	12.4	0.002
- Performing clinical procedures	15	10%		
Confidence in Educating Pregnant Women				
- Strongly agree/Agree	128	85%	8.6	0.013
- Neutral/Disagree	22	15%		
Confidence in Providing Prenatal Care				
- Strongly agree/Agree	113	75%	6.2	0.045
- Neutral/Disagree	37	25%		

Table 3 reveals that lack of awareness was the most common barrier (40%; *χ*^2^ = 15.8, *p* = 0.001), followed by financial limitations (25%; *χ*^2^ = 9.2, *p* = 0.027) and cultural beliefs (20%; *χ*^2^ = 7.5, *p* = 0.023). Accessibility related barriers were reported by 15% of participants; however, this finding did not reach statistical significance (*χ*^2^ = 5.1, *p* = 0.078).

**Table 3:** Barriers to delivering oral care.

Barrier	Quantitative Frequency (%)	Chi-square (χ^2^)	*p*-value	Qualitative Themes
Lack of Awareness	60 (40%)	15.8	0.001	"Myths about dental safety during pregnancy are common."
Financial Constraints	38 (25%)	9.2	0.027	"Cost and insurance issues prevent access."
Cultural Beliefs	30 (20%)	7.5	0.023	"Families discourage dental visits during pregnancy."
Accessibility Issues	22 (15%)	5.1	0.078	"Rural areas lack dental clinics."

A notable disparity was observed in relation to training (*χ*^2^ = 25.3, *p* < 0.001), with 60% of respondents indicating that they had not received formal training in maternal and child oral health. Despite this gap, a large proportion (80%) expressed the view that such training should be mandatory (*χ*^2^ = 18.7, *p* < 0.001), suggesting a strong perceived need for structured skill development (Table 4).

**Table 4:** Training impact on confidence.

Training Received?	High Confidence (*n* = 113)	Low Confidence (*n* = 37)	χ^2^	*p*-value	Qualitative Excerpt
Yes (*n* = 60)	78%	22%	9.45	0.002*	"PG training taught me counseling skills."
No (*n* = 90)	55%	45%			"I avoid pregnant patients—no formal guidance."

*p* < 0.05 was considered statistically significant.

Differences between rural and urban settings were also evident. A lack of patient awareness was reported more frequently in rural areas (68%) compared to urban settings (45%) (*χ*^2^ = 4.32, *p* = 0.038). Similarly, financial constraints were commonly noted among rural participants (41%; *χ*^2^ = 6.84, *p* = 0.009), and cultural myths were reported by 36% in rural areas vs. 18% in urban settings (*χ*^2^ = 5.12, *p* = 0.024). Accessibility emerged as the most prominent barrier in rural regions, affecting 59% of participants, whereas only 12% of urban respondents reported similar concerns (*χ*^2^ = 25.7, *p* < 0.001).

Participants who had received formal training demonstrated higher levels of confidence (78%) compared with those without training (55%). Conversely, lower confidence was more frequently observed among untrained participants (45% vs. 22%). This association was statistically significant (*χ*^2^ = 9.45, *p* = 0.002).

A multivariable binary logistic regression analysis was used to find independent characteristics related to high confidence in providing maternal oral health care. The model demonstrated good fit (Hosmer–Lemeshow *χ*^2^ = 6.82, *p* = 0.56) and explained 42% of the variance in confidence (Nagelkerke R^2^ = 0.42), with an overall classification accuracy of 78.5%.

After adjustment for professional and practice-related characteristics, formal training in maternal and child oral health emerged as the strongest predictor of high confidence. Participants who had received formal training were significantly more likely to report high confidence compared with those without training (adjusted OR=3.46; 95% CI: 1.62–7.38; *p* = 0.001). Greater professional experience was also associated with increased confidence (adjusted OR=1.95; 95% CI: 1.11–3.44; *p* = 0.020). Participation in community outreach activities was another significant predictor, with higher odds of reporting high confidence among those involved in outreach programs (adjusted OR=2.29; 95% CI: 1.13–4.65; *p* = 0.021). In contrast, practising in rural settings was associated with lower odds of high confidence compared with urban practice settings (adjusted OR=0.40; 95% CI: 0.18–0.90; *p* = 0.026). Education level (faculty vs. postgraduate trainee) showed a positive but statistically non-significant association with confidence (adjusted OR=1.79; 95% CI: 0.94–3.41; *p* = 0.079) (Table 5).

**Table 5:** Multivariable binary logistic regression analysis identifying predictors of high confidence in providing maternal and child oral health care among public health dentists (*n* = 150).

Predictor	B (Coefficient)	S.E.	Wald χ^2^	df	*p*-value	Odds Ratio (Exp B)	95% C.I. for OR
Training Received	1.24	0.38	10.56	1	0.001	3.46	1.62–7.38
Years of Experience	0.67	0.29	5.38	1	0.020	1.95	1.11–3.44
Practice Setting (Rural)	−0.92	0.41	4.98	1	0.026	0.40	0.18–0.90
Outreach Participation	0.83	0.36	5.31	1	0.021	2.29	1.13–4.65
Education Level (Faculty)	0.58	0.33	3.09	1	0.079	1.79	0.94–3.41
Constant	−1.15	0.45	6.53	1	0.011	0.32	-

B, Logistic regression coefficient; S.E., Standard Error; Wald χ^2^, Wald Chi-square statistic; df, Degrees of Freedom; OR, Odds Ratio; CI, Confidence Interval.

*p* < 0.05 is considered statistically significant.

### 3.2 Qualitative findings

**Figure 2:**
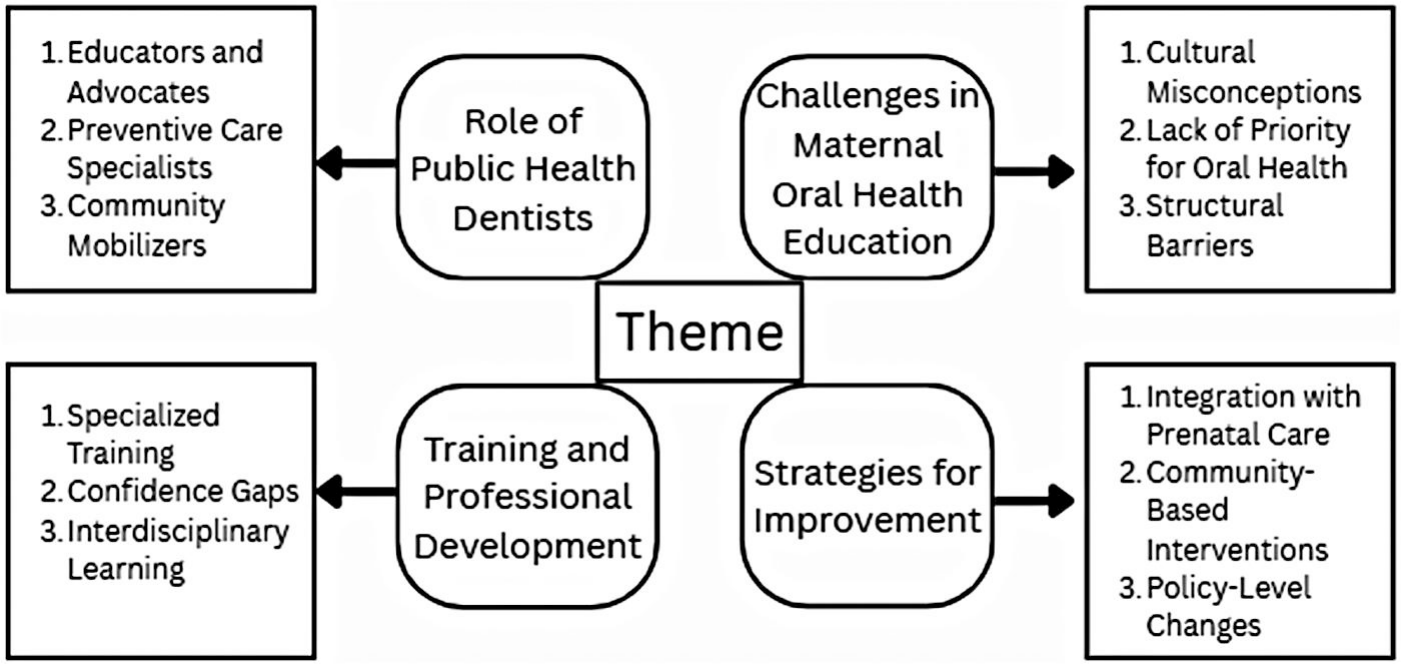
The four major themes, along with the sub-themes identified during qualitative analysis.

The Qualitative content analysis yielded four major themes (Figure 2): (1) Role of Public Health Dentists, (2) Challenges in maternal oral health education, (3) Training and professional development and (4) Strategies for improvement. These themes provided depth to the quantitative findings.

#### 3.2.1 Theme 1: the role of public health dentists

**Figure 3:**
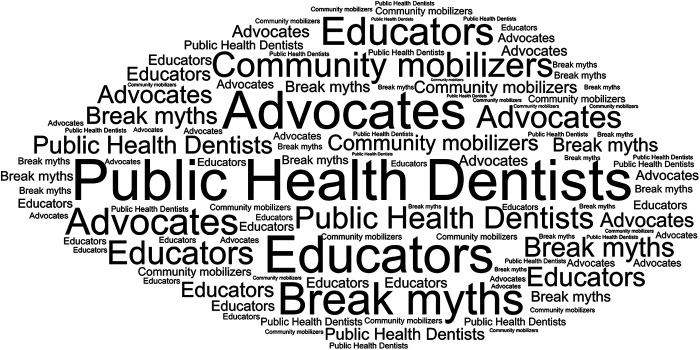
Most frequently reported responses by the participants about the role of public health dentists.

Thematic analysis revealed that Public Health Dentists primarily viewed themselves as educators, advocates and community mobilizers, with most participants emphasising preventive and promotional responsibilities over clinical treatment (Figure 3). Participants frequently described their primary roles as providing oral health education to pregnant women, particularly in rural areas where cultural beliefs and misinformation were widely prevalent.

One respondent explained, “Our main job is to educate mothers- many don't realise gum disease can lead to preterm birth” (Respondent 3). Another participant emphasised collaboration with frontline workers, stating “In villages, we work with Accredited Social Health Activist (ASHA) workers to break myths like dental treatment harms the baby” (Respondent 7). Preventive activities, including fluoride-based programs and oral hygiene awareness sessions, were commonly reported. These findings suggest that Public Health Dentists largely contribute through community-oriented education and anticipatory guidance, rather than direct clinical interventions.

#### 3.2.2 Theme 2: challenges in maternal and oral health education

**Figure 4:**
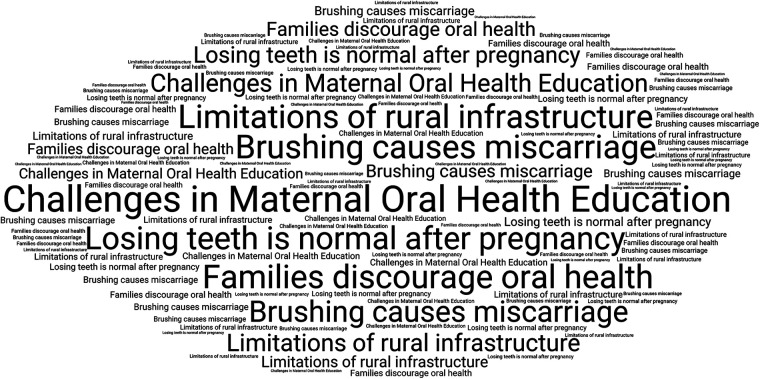
Commonly reported challenges in maternal oral health education among participants.

Participants described a range of challenges in delivering maternal oral health education, particularly those related to deeply rooted cultural beliefs, low prioritisation of dental care, and structural barriers such as inadequate rural infrastructure (Figure 4). Several respondents referred to the persistence of common myths, including the belief that brushing or undergoing dental treatment during pregnancy may be harmful. One participant stated, “Many believe losing teeth is normal after pregnancy-we must correct this” (Respondent 2).

Women from lower-income households were often perceived to prioritise general health concerns over dental needs. Limited availability of dental services, along with transportation difficulties in rural areas, were also frequently mentioned. In addition, family influence appeared to play a significant role in care-seeking behaviour. As one respondent explained, “Even when free camps are held, women don't come because families discourage it” (Respondent 5). These observations suggest the need for culturally sensitive and community-oriented strategies to address both perceptual and structural barriers.

#### 3.2.3 Theme 3: training and professional development

**Figure 5:**
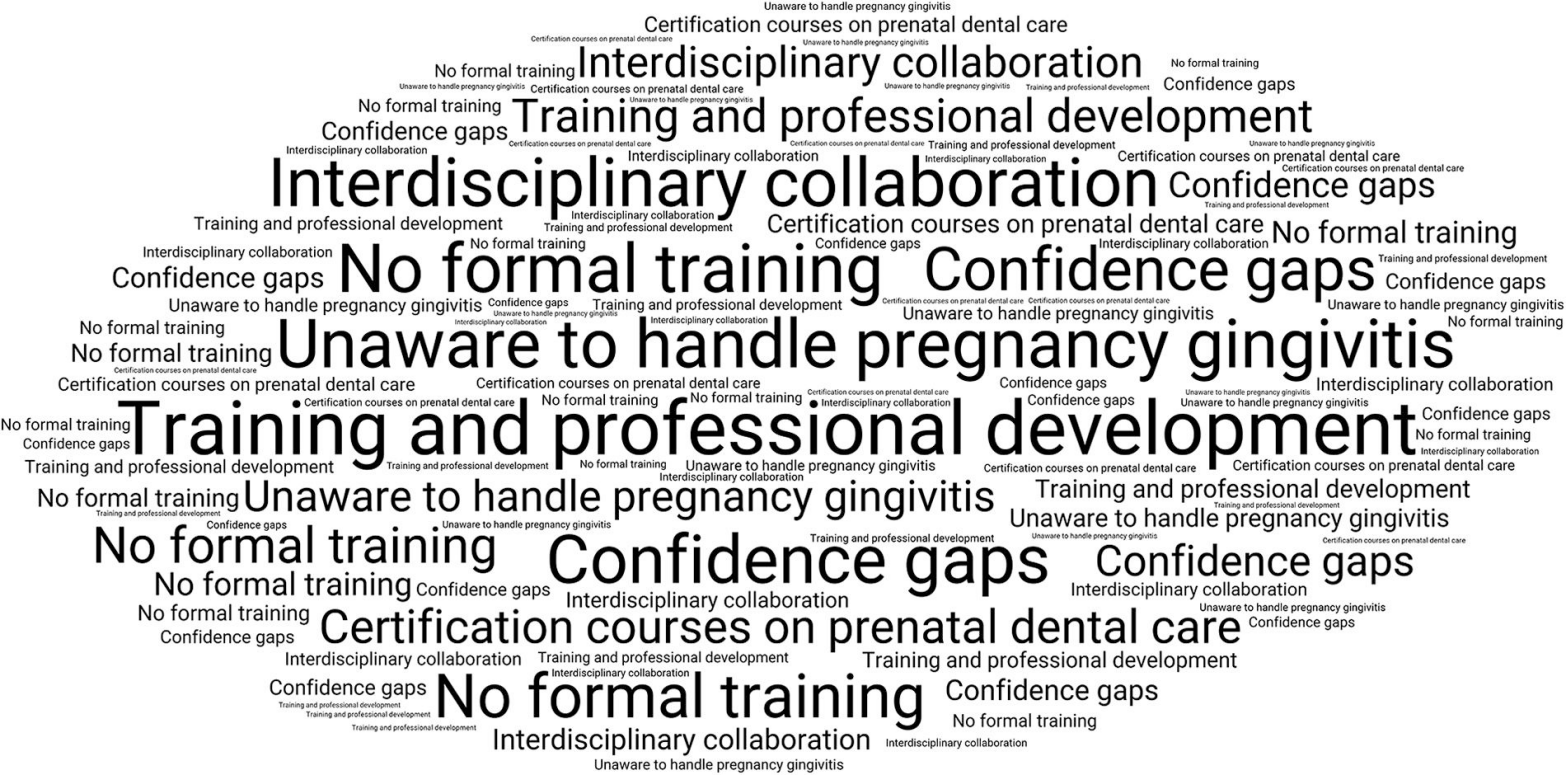
Frequently reported responses related to training and professional development needs.

Findings from the analysis indicated notable gaps in formal training and professional preparation related to maternal and child oral health (Figure 5). A majority of participants (60%) reported not having received structured training, and several expressed feeling insufficiently prepared to manage pregnancy-related oral health conditions. One participant remarked, “I learned communication skills in post-graduation, but I did not learn how to handle pregnancy gingivitis” (Respondent 1). Limited hands-on exposure appeared to contribute to reduced confidence, and participants frequently mentioned the need for closer interdisciplinary collaboration with obstetricians.

Participants also suggested the introduction of more structured learning opportunities, including certification programs and continuing education initiatives. As one respondent stated, “We need certification courses on prenatal dental care” (Respondent 6). Overall, these observations point towards the importance of developing competency-based and practice-oriented training approaches to enhance workforce preparedness.

#### 3.2.4 Theme 4: strategies for improvement

**Figure 6:**
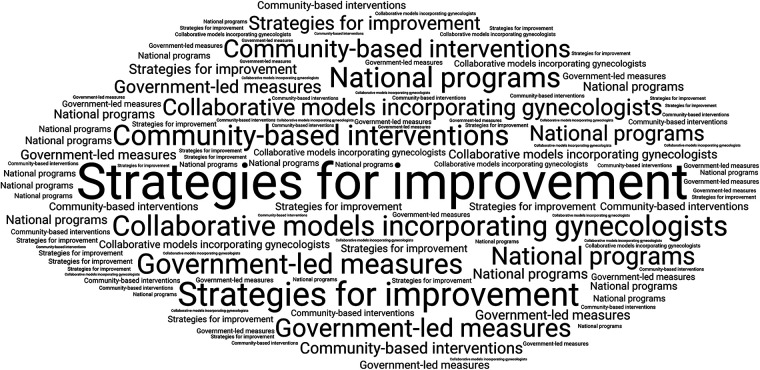
Participant-reported strategies for improving maternal and child oral health services.

Participants outlined a range of multi-level strategies aimed at strengthening maternal and child oral health services, with emphasis on clinical integration, community engagement, and supportive policy measures (Figure 6). A commonly suggested approach was the inclusion of oral health assessments within routine antenatal care. As one respondent noted, “If blood pressure is checked during pregnancy, why not oral health?” (Respondent 4). Community-based initiatives, particularly those involving mother-child groups, schools, and frontline health workers, were frequently considered important for addressing cultural misconceptions and improving oral health literacy.

At the policy level, numerous participants advocated for structured referral mechanisms and government-mandated oral screenings during pregnancy. One respondent remarked, “We need a national program linking dentists to maternity hospitals” (Respondent 9). Taken together, these perspectives suggest strong support for a more integrated and interdisciplinary approach, reinforced by appropriate policy-level interventions, to improve maternal and child oral health service delivery.

The survey found that Public Health Dentists largely focus on preventive and educational responsibilities, with high confidence in maternal and oral health services. Lack of knowledge, financial constraints, and cultural views were identified as key impediments, especially in rural areas. Formal training significantly increased professional confidence, while qualitative findings highlighted the importance of integrated, culturally responsive, and policy-supported measures to improve mother and child oral health services.

### 3.3 Mixed-methods integration of urban–rural disparities

To improve integration of the quantitative and qualitative findings, a joint display table was created to link statistically significant urban-rural differences with relevant qualitative excerpts (Table 6). This approach allowed numerical results to be interpreted alongside participants' experiences.

**Table 6:** Mixed-methods joint display synthesizing statistically significant urban–rural differences in reported barriers, supported by qualitative narratives.

Quantitative Finding	Urban (*n* = 128)	Rural (*n* = 22)	χ^2^ (*p*-value)	Supporting Qualitative Quote	Integrated Interpretation
Lack of Awareness	45%	68%	4.32 (0.038*)	“Rural women rely on traditional healers more than urban ones.”	Rural participants reported significantly higher awareness gaps, supported by qualitative accounts of misinformation and reliance on traditional practices.
Financial Barriers	20%	41%	6.84 (0.009*)	“No subsidies for dental care in villages.”	Quantitative disparity is reinforced by qualitative perceptions of limited financial protection in rural areas.
Cultural Myths	18%	36%	5.12 (0.024*)	“Extracting teeth=blindness belief persists.”	Statistical differences align with narratives describing entrenched cultural misconceptions in rural communities.
Clinic Accessibility	12%	59%	25.7 (<0.001*)	“Nearest dental clinic is 50 km away.”	Strong quantitative disparity is supported by qualitative descriptions of infrastructural and geographic barriers.

*p* < 0.05 was considered statistically significant.

Integrated analysis found that reported barriers differed significantly across urban and rural areas, as reflected in comparable qualitative narratives. Rural respondents reported significantly higher rates of barriers to awareness, financial constraints, cultural views, and clinic accessibility. These differences were further explained by qualitative perspectives describing misinformation, reliance on traditional practices, financial constraints, and challenges related to geographic access to services. Taken together, the findings suggest that rural practice settings may face multiple overlapping challenges in delivering maternal and child oral health services.

## 4 Discussion

### 4.1 Role of public health dentists

This mixed-method study identified that Public Health Dentists predominantly functioned as educators and preventive-health advocates rather than clinicians. These findings highlight an underutilised but critical workforce that, with improved support from organised training and system-level reforms, may improve mother and child oral health outcomes.

The findings of this study indicate that Public Health Dentists largely function in preventive and educational roles. This aligns with existing literature, which describes Public Health Dentistry as being primarily focused on community-based prevention rather than curative care (11, 12). The emphasis on counseling and outreach observed in the present study also appears similar to patterns reported in low-and middle-income countries (LMICs), where Public Health Dentists often address limitations in clinical infrastructure through community-level engagement and mobilisation (13, 19). These findings were supported by qualitative narratives, where participants consistently described their roles as educators and community mobilisers, particularly through community outreach and collaboration with frontline health workers such as ASHA workers. Together, these findings suggest that while Public Health Dentists play a key preventive role, their clinical potential within maternal healthcare systems remains underutilised.

### 4.2 Barriers to MCOH service delivery

Participants from rural areas reported a higher burden of barriers, including limited awareness, financial constraints, cultural misconceptions, and restricted access to dental services. Similar patterns have been described in studies from low- and middle-income countries (LMICs), where rural populations often experience additional challenges related to inadequate infrastructure and lower health literacy (13, 15). The consistency between the present findings and existing literature suggests that comparable structural barriers may be present across similar settings, highlighting the need for context-specific strategies in rural areas (16, 21).

These quantitative findings were further supported by qualitative insights, where participants described persistent myths regarding dental care during pregnancy, low prioritisation of oral health, and the influence of family members on care-seeking behaviour. Structural challenges such as limited service availability and transportation barriers were also frequently reported. The convergence of quantitative and qualitative findings indicates that both structural and socio-cultural barriers significantly influence service delivery. In addition, participants in this study frequently referred to the role of Accredited Social Health Activist (ASHA) and Anganwadi workers. This observation is supported by previous evidence indicating that community health workers play an important role in improving maternal and child oral health uptake, particularly among underserved populations (11, 13, 17).

### 4.3 Training and professional development

A key finding of the study was the significant lack of formal MCOH training and related low clinical confidence, despite global recommendations confirming the safety and relevance of dental care during pregnancy (22). This finding is consistent with earlier research that found dentists were hesitant due to insufficient training, legal concerns, and fear of complications (21, 23). However, our findings differ from those in countries with structured maternal oral health training programs, such as the Detroit Mercy Model, where specialised modules dramatically enhanced knowledge and abilities (24). This difference may be explained by India's limited incorporation of MCOH into dental curricula and the scarcity of opportunities for hands-on experience during postgraduate studies.

The multivariable regression analysis pointed to the importance of training and professional exposure in influencing confidence among Public Health Dentists. Those who had received formal training in maternal and child oral health (MCOH) were more than three times as likely to report higher confidence compared with those without such training. The strong statistical association between training and confidence was further reinforced by qualitative insights highlighting gaps in clinical preparedness. These quantitative findings were further reflected in the qualitative data, where participants described how postgraduate training and practical experience contributed to better communication and patient management. One respondent noted, “PG training taught me how to talk to pregnant women and address their fears” (Respondent 3). Another participant shared, “In my early years, I avoided pregnant patients. Now I handle them comfortably” (Respondent 8).

Participation in community outreach programs was also associated with higher professional confidence, with dentists involved in such activities reporting more than twice the likelihood of high confidence. Participants in the interviews discussed how outreach exposure offers practical learning experiences beyond formal education. Together, these findings emphasise the importance of competency-based training, practical exposure, and continuing professional development in improving workforce readiness.

### 4.4 Rural–urban disparities

Lower confidence levels were more commonly reported among rural practitioners compared with those working in urban settings. Similar concerns emerged from the qualitative data, particularly regarding structural limitations such as restricted referral networks and limited access to specialist support.

Mixed-method integration revealed that rural practitioners face compounded barriers, as supported by both statistically significant differences and contextual qualitative accounts. According to one participant, “In villages, we lack backup—no specialists to refer to if complications arise.” (Respondent number five). These findings highlight the additional challenges faced in rural settings, including accessibility issues and limited healthcare infrastructure. These findings suggest that rural–urban disparities are multifactorial and require targeted, context-specific interventions to improve service delivery.

### 4.5 System-level implications

Interprofessional collaboration was identified as an important gap in the present study. Similar observations have been reported in the literature, where prenatal care providers are often unlikely to refer pregnant women for dental assessment despite existing clinical recommendations (25). This gap may be linked to fragmented health systems, limited cross-disciplinary training, and the absence of well-defined referral pathways, all of which can delay the integration of oral health into routine maternity care.

The results of the present study are in line with international efforts at integrating oral health into maternal healthcare services, as reflected in policy models from countries such as Brazil and South Africa, as well as recommendations from global health organisations (12–14). In contrast, India currently does not have similarly structured guidelines for the integration of maternal oral health. Improving coordination between national oral health initiatives and maternal health programs may strengthen service delivery within primary care settings. Maternal oral health has a considerable impact on child outcomes, such as early childhood caries and adverse birth outcomes (3, 26). The low service utilisation in our study is consistent with international data, such as the Centres for Disease Control and Prevention's (CDC) Pregnancy Risk Assessment Monitoring System (PRAM) findings, which show that only 23%–43% of pregnant women receive dental care (17). This closeness shows that structural, cultural, and professional impediments to maternal oral healthcare exist beyond national borders and necessitate a coordinated global and national response.

The National Oral Health Programme (NOHP) places strong emphasis on prevention, oral health promotion, and strengthening workforce capacity (27). However, its integration with maternal health services in practice appears to be limited. While the preventive and educational roles reported by participants are broadly aligned with the objectives of the National Oral Health Programme (NOHP), several gaps remain. In particular, issues related to formal training, interprofessional collaboration, and the absence of structured referral pathways indicate ongoing implementation challenges within maternal and child health settings. Furthermore, limited coordination between NOHP and national maternal health initiatives such as RMNCH + A may partly explain the underutilisation of Public Health Dentists at the primary care level.

In the context of these policy gaps, the findings of this study suggest that the role of Public Health Dentists within India's maternal and child health system could be strengthened, particularly through better training and improved collaboration with other healthcare professionals. Introducing routine prenatal oral screening within national oral health programs may allow earlier identification of oral health needs. In addition, integrating interprofessional education into both medical and dental training, along with developing clearer frameworks within RMNCH + A, could improve coordination of care (12, 28). Existing outreach systems, such as primary healthcare providers, ASHA workers, and Anganwadi centres, may also play an important role. Approaches like training-of-trainers and community-based engagement strategies could help expand services more effectively, especially in underserved areas (11, 13). Overall, these measures could help address current gaps and support improvements in maternal and child oral health across India.

### 4.6 Implications

To further clarify the contribution of the study, the implications are presented in terms of theoretical and practical relevance.

#### 4.6.1 Theoretical implications

**Figure 7:**
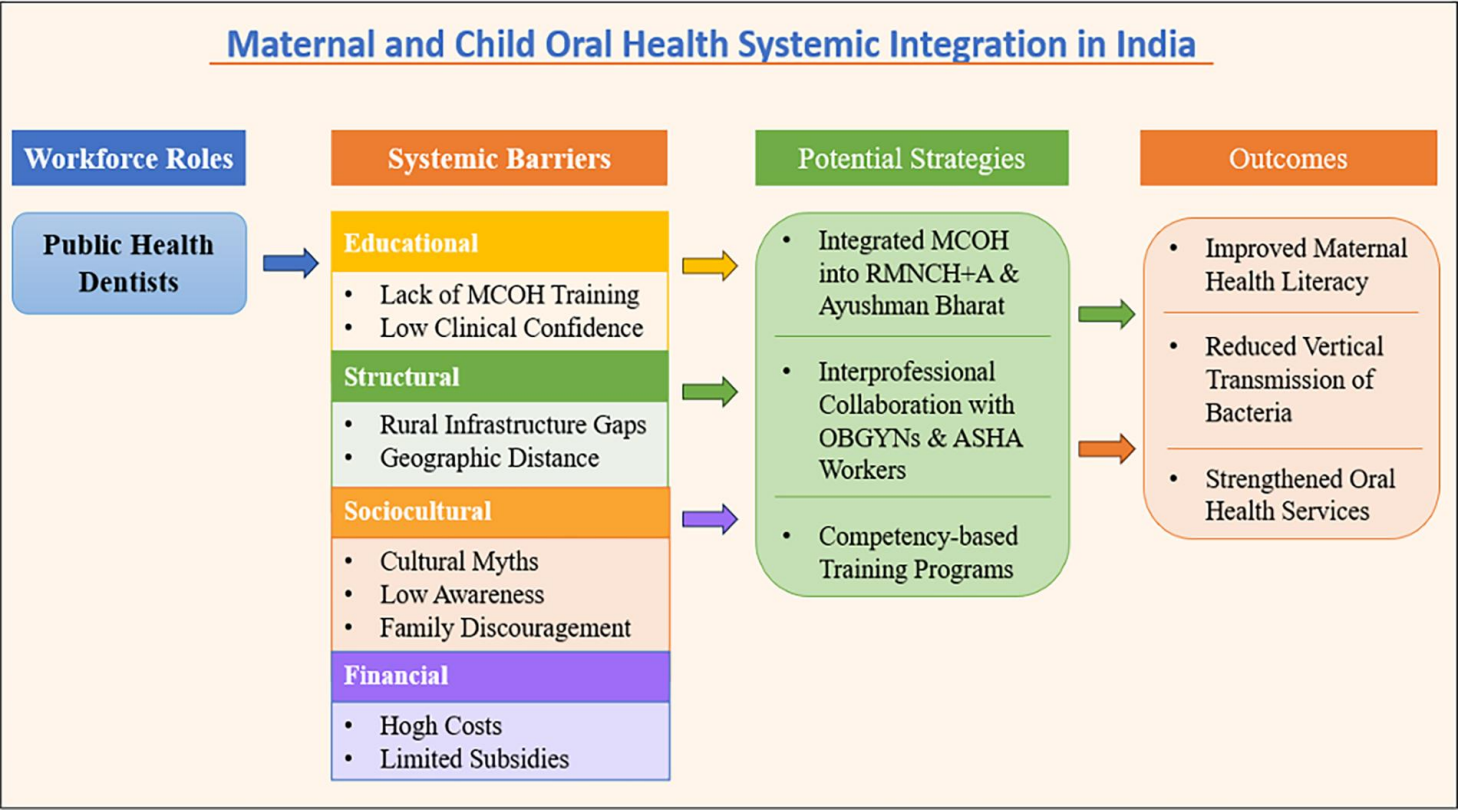
Conceptual framework illustrating the interaction between workforce roles, systemic barriers, and potential strategies influencing maternal and child oral health service delivery in India.

Beyond descriptive findings, this study provides an analytical understanding of maternal and child oral health service delivery by conceptualising how workforce roles, systemic barriers, and strategic interventions interact to influence service delivery within the health system. The conceptual framework developed in this study (Figure 7) synthesises these findings into interconnected domains of workforce roles, systemic barriers, potential strategies, and service delivery outcomes. The framework illustrates how system-level constraints influence workforce capacity and, in turn, affect service accessibility and quality. It also highlights how targeted strategies, including training, policy integration, and interprofessional collaboration, may improve maternal oral health literacy and strengthen service delivery. Importantly, the model may be applicable to other low- and middle-income settings facing similar health system constraints, thereby extending the relevance of the findings beyond the immediate study context.

#### 4.6.2 Practical and policy implications

The integration of quantitative and qualitative findings strengthens the validity of the results by providing both statistical evidence and contextual understanding of workforce challenges. The findings highlight several actionable strategies, including integrating oral health screening into routine antenatal care, strengthening referral pathways, improving interprofessional collaboration, and expanding competency-based training programs. Existing platforms such as Ayushman Bharat Health and Wellness Centres, along with ASHA and Anganwadi networks, may provide opportunities for implementation (29). Collectively, these strategies support a more integrated and system-oriented approach to improving maternal and child oral health outcomes in India.

### 4.7 Strengths and limitations

This study adds to the limited mixed-methods research examining the role of Public Health Dentists in maternal and child oral health (MCOH) in India. Bringing together quantitative and qualitative findings helped provide a more detailed understanding of workforce roles, capabilities, and challenges. At the same time, a few limitations should be considered. The use of convenience sampling and reliance on self-reported data may introduce bias, and the geographical spread of participants was limited. The findings of the study should be interpreted as perceptions of participating Public Health Dentists rather than nationally representative estimates. In addition, the absence of detailed state- or region-wise data limits the ability to assess geographic variability in workforce distribution and may affect the generalisability of findings across different regions of India. The findings of the study should be interpreted as perceptions of participating Public Health Dentists rather than nationally representative estimates.

It is also important to note that a large proportion of participants (80%) were postgraduate trainees in Public Health Dentistry. Consequently, the findings predominantly reflect the perspectives of early-career, academically oriented professionals, which may differ from those of more experienced practitioners working in diverse clinical and administrative settings. Early-career professionals may place greater emphasis on preventive and educational roles, while potentially having limited exposure to system-level challenges such as policy implementation, resource allocation, and long-term program management. This may influence the interpretation of workforce roles and barriers reported in the study.

### 4.8 Future directions

Future research could focus on understanding the long term-impact of MCOH training programs, integrated referral systems, and related policy changes. It would also be useful to examine cost-effectiveness and explore newer approaches, such as tele-dentistry, particularly in rural and underserved settings. These directions may help in developing more practical and sustainable models for maternal and child oral healthcare.

## 5 Conclusion

This study demonstrates that Public Health Dentists primarily perform preventive and educational responsibilities in mother and child oral health settings, but they face significant training and systemic barriers. The findings indicate that targeted training initiatives, more interprofessional collaboration, and better incorporation of oral health into existing maternal health programs could help address identified service gaps. Further longitudinal and implementation-based research is warranted to evaluate the effectiveness and scalability of such approaches.

## Data Availability

The original contributions presented in the study are included in the article/Supplementary Material, further inquiries can be directed to the corresponding authors.
