# Supplementary material for: Public health dentists in maternal and child oral health in India: workforce roles, systemic barriers, and policy implications from a mixed-methods study
Source: Front Oral Health. 2026 May 12;7:1839520. doi: 10.3389/froh.2026.1839520 (PMC13201444; doi:10.3389/froh.2026.1839520)
Supplement: Supplementary file 1 [file Supplementaryfile1.docx]

The Role of Public Health Dentists in Maternal and Child Oral Health

**Demographic Details**:

**Name** -

**Gender -**

**Age** -

**Profession** –

1. 1^st^ Year Postgraduate Student
2. 2^nd^ Year Postgraduate Student
3. 3^rd^ Year Postgraduate Student
4. Faculty / Public Health Dentist

**Years of experience -**

**Place of practice** - Urban/Rural

**Involvement in public health programs** - Yes/No

# Questionnaire:

1. What is the primary role of a public health dentist in maternal and child oral health?

A) Performing root canal and crown procedures for mothers and children

B) Designing and implementing community-based preventive oral health programs for mothers and children

C) Providing cosmetic dental services to pregnant women and children

D) Running specialty orthodontic practices for infants and expecting mothers

2. Why is it important for pregnant women to receive oral health education?

A) Because pregnancy permanently weakens teeth and leads to inevitable decay

B) To treat minor aesthetic changes in the gingival tissue

C) To discourage any dental treatment during pregnancy

D) To prevent periodontal disease, which is linked to preterm birth

3. What role does a public health dentist play in reducing pregnancy-related gingivitis?

A) Advising pregnant women to avoid brushing if there is gingival bleeding

B) Recommending herbal remedies instead of dental check-ups

C) Providing professional cleaning (scaling) and oral hygiene education

D) Delaying any dental care until after childbirth

4. Why should a public health dentist screen for periodontal disease in pregnant women?

A) Because pregnancy hormones eliminate periodontal disease risk

B) Because oral health has no connection with pregnancy outcomes

C) Because periodontal disease can contribute to preterm birth and low birth weight

D) Because dental examinations are not advisable during pregnancy

5. Which of the following is an appropriate fluoride recommendation for a pregnant woman?

A) Fluoride should be avoided entirely during pregnancy

B) Fluoride use should begin only after delivery

C) Fluoride mouthrinse should replace toothbrushing during pregnancy

D) Continue using fluoridated toothpaste and drinking fluoridated water as part of your daily routine

6. What strategy can a public health dentist use to improve access to oral health care for pregnant women?

A) Focus oral health efforts only on children, not mothers

B) Delay dental care until after childbirth

C) Integrate oral health check-ups and referrals into routine prenatal visits

D) Provide services only in tertiary urban hospitals

7. How can a public health dentist contribute to preventing early childhood caries in newborns?

A) Wait until the child starts school to begin oral health education

B) Educate expectant mothers on oral hygiene and sugar intake during pregnancy

C) Encourage sugary snacks for infants to stimulate chewing

D) Recommend avoiding fluoride use in early childhood

8. Which of these is an effective way by which public health dentists can educate parents about early childhood oral health?

A) Limit education to online resources only

B) Conduct awareness campaigns and community-based workshops

C) Restrict oral health messages to private practice settings

D) Avoid educational efforts until children have permanent teeth

9. What is the ideal timing for a child’s first dental visit, as recommended by public health dentists?

A) At birth

B) At 5 years of age

C) By the first birthday or within 6 months of the first tooth eruption

D) When the child begins school

10. What public health measure helps prevent dental diseases in pregnant women and children?

A) Community water fluoridation

B) Recommending brushing without toothpaste

C) Promoting emergency-only dental visits

D) Avoiding all fluoride use during pregnancy

11. How does early intervention by public health dentists benefit children's overall health?

A) Reduces the risk of systemic diseases linked to poor oral health

B) Encourages children to skip regular dental visits

C) Promotes a sugar-rich diet to strengthen teeth

D) Delays oral health education until adolescence

12. How can public health dentists support school-based oral health programs?

A) Promote sugary drinks in school canteens

B) Provide fluoride varnish applications and dental screenings

C) Limit care to students with visible decay only

D) Focus only on staff oral health, not children’s

13. Which program is most relevant for integrating oral health care into maternal health services?

A) Cardiovascular Disease Prevention Program

B) Childhood Immunization Program

C) Maternal and Child Health Program

D) National Diabetes Prevention Program

14. Why is collaboration between public health dentists and government health programs important for early childhood care?

A) It facilitates timely, preventive care access for underserved mothers and children

B) It excludes dental screenings from routine child health visits

C) It promotes delaying dental care until school age

D) It restricts early dental interventions to emergency cases only

15. Which of the following practices helps prevent vertical transmission of cariogenic bacteria from mother to child?

1. Ignoring maternal oral hygiene during pregnancy
2. Giving the infant sweetened pacifiers or feeding bottles at bedtime
3. Avoid sharing spoons or cleaning pacifiers with the mother’s mouth
4. Promote dental visits for both mother and child, only in case of emergency

16. Public health dentists should educate mothers about the risk of vertical transmission of Streptococcus mutans.

1. Strongly agree
2. Agree
3. Neutral
4. Disagree

E) Strongly Disagree

17. I feel confident educating pregnant women about oral health.

1. Strongly agree
2. Agree
3. Neutral
4. Disagree
5. Strongly Disagree

18. I feel confident in providing prenatal dental care.

A) Strongly agree
B) Agree
C) Neutral
D) Disagree

E) Strongly Disagree

19. Do you think it is important to advise pregnant women on the use of tobacco and its ill effects on the child?

A) Yes

B) No

C) Maybe

20. Have you ever participated in a maternal and child oral health program or outreach activity?

A) Yes

B) No

21. Did you undergo any formal training program for maternal and child oral health?

A) Yes

B) No

If yes, then specify the program: ____________________________________________

22. According to you, what are the perceived barriers in delivering oral care to pregnant women in the community?

_______________________________________________________________________
