# Supplementary material for: Public health dentists in maternal and child oral health in India: workforce roles, systemic barriers, and policy implications from a mixed-methods study
Source: Front Oral Health. 2026 May 12;7:1839520. doi: 10.3389/froh.2026.1839520 (PMC13201444; doi:10.3389/froh.2026.1839520)
Supplement: Supplementary file 2 [file Supplementaryfile2.docx]

**Qualitative questionnaire**

1. What do you believe is the most important role of a public health dentist in maternal and child oral health?
2. In your experience, how receptive are pregnant women to oral health education, and what factors influence their response?
3. Can you describe any challenges you've faced while trying to educate expectant mothers about oral hygiene practices?
4. How can community-based interventions be improved to better support oral health in mothers and young children?
5. What are your thoughts on integrating oral health services into routine prenatal care?
6. How has your postgraduate training (or lack thereof) influenced your confidence in providing prenatal oral health education and care?
7. What are the most common misconceptions pregnant women have about oral health, based on your observations?
8. In your opinion, what role does collaboration between dentists and other healthcare providers play in maternal oral health outcomes?
9. What strategies have you found most effective in educating communities about the prevention of early childhood caries?
10. Have you received any formal training for maternal and child oral health, and do you think training programs should be made compulsory for public health dentists for maternal and child oral health?
11. According to your experience, what are the key barriers in delivering oral healthcare to underserved pregnant women in rural areas?
